# The Association Between Sleep and Metabolic Syndrome: A Systematic Review and Meta-Analysis

**DOI:** 10.3389/fendo.2021.773646

**Published:** 2021-11-19

**Authors:** Tingting Che, Cheng Yan, Dingyuan Tian, Xin Zhang, Xuejun Liu, Zhongming Wu

**Affiliations:** ^1^ NHC Key Laboratory of Hormones and Development, Tianjin Key Laboratory of Metabolic Diseases, Chu Hsien-I Memorial Hospital & Tianjin Institute of Endocrinology, Tianjin Medical University, Tianjin, China; ^2^ Department of Neurology, Chu Hsien-I Memorial Hospital, Tianjin Medical University, Tianjin, China

**Keywords:** sleep duration, metabolic syndrome, meta-analysis, obesity, diabetes

## Abstract

**Purpose:**

Sleep duration is thought to play a key role in the development of metabolic syndrome. However, the results have been inconsistent.

**Methods:**

We conducted a systematic review and meta-analysis of cohort studies and searched publications in PubMed, Embase, Cochrane Central Register of Controlled Trials, and Clinicaltrials.gov. The summary relative risks (RRs) were estimated using a random model. The sensitivity analysis was performed by sequentially excluding each study to test the robustness of the pooled estimates.

**Finding:**

We included 13 studies involving 300,202 patients in which short sleep and long sleep significantly increased the risk of metabolic syndrome 15% (RR = 1.15, 95%CI = 1.09-1.22, p < 0.001) and 19% (RR = 1.19, 95%CI = 1.05-1.35, p < 0.001). Moreover, the relationship between sleep duration and metabolic syndrome risk presented a U-shaped curve. Short and long sleep increased the risk of obesity by 14% (RR = 1.14, 95%CI = 1.07-1.22, p<0.001) and 15% (RR = 1.15, 95%CI = 1.00-1.30, p = 0.04), and high blood pressure 16% (RR = 1.16, 95%CI = 1.02-1.31, p = 0.03) and 13% (RR = 1.13, 95%CI = 1.04-1.24, p = 0.01), respectively. Short sleep can potentially increase the risk of high blood sugar by 12% (RR = 1.12, 95%CI = 1.00-1.15, P = 0.05).

**Implications:**

Based on our findings, sleep is a behavior that can be changed and is economical. Clinically doctors and health professionals should be encouraged to increase their efforts to promote healthy sleep for all people.

## 1 Introduction

Metabolic syndrome (MS) is a collection of metabolic disorders, including obesity, hypertension, hypertriglyceridemia, low high-density lipoprotein (HDL) cholesterol, and hyperglycemia. Prevalence of MS ranges from 20 to 45%, and the incidence of MS is projected to increase to approximately 53% by 2035 (1). The syndrome leads to adverse cardiovascular events (2) and the spread of diseases such as cancer (3). The total cost of these diseases, including the potential loss of health care and economic activity, places a significant financial burden on patients and health systems (4). Therefore, it is important to identify modifiable risk factors for MS (5).

Recently, the relationship between sleep and metabolic syndrome has attracted wide attention. Epidemiological evidence has reported the relationship between sleep duration and MS. However, the information published was not consistent (6–8). Ju (6) included 11 cross-sectional studies and three cohort studies and concluded that both short sleep and long sleep are risky behaviors that increase the risk of metabolic syndrome. Another meta-analysis, consisting of 10 cross-sectional studies and two cohort studies, concluded that short sleep duration was associated with metabolic syndrome development, while long sleep duration was not (7). The final meta-analysis, which included 18 cross-sectional studies, found a dose-response relationship between short sleep duration and metabolic syndrome. However, it does not support the idea that long sleep is associated with metabolic syndrome (8). Summing up previous meta-analyses, it can be found that short sleep is associated with an increased risk of MS, but the relationship between long sleep and MS risk is still controversial.

Wang (9) found a U-shaped relationship between sleep duration and the risk of hypertension. Shan (10) reported a U-shaped relationship between sleep duration and the risk of diabetes. Hypertension and diabetes are components of metabolic syndrome. Therefore, we hypothesize that a U-shaped relationship between sleep duration and the risk of metabolic syndrome exists as well. At the same time, we found that many cohort studies on sleep duration and metabolic syndrome have emerged in recent years. Given that cohort studies possess a higher level of evidence and can also draw causal inferences, we mainly included the latest cohort studies for our meta-analysis.

## 2 Methods

### 2.1 Literature and Search Strategy

We searched four databases (PubMed, Embase, Cochrane Central Register of Controlled Trials, and Clinicaltrials.gov) for reports relating to sleep and MS. The combination of two sets of keywords and exploded controlled vocabulary terms is showed in **Table S1**. The literature search was limited to English, updated to October 18, 2021. The protocol was registered in PROSPERO (No.CRD42020222990).

### 2.2 Inclusion Criteria and Data Extraction

The studies included in the meta-analysis must have met all the following inclusion criteria: 1) Outcome variables need to be defined as the incidence of MS or a group of metabolic abnormalities, including hyperglycemia, obesity, hypertension, or dyslipidemia. 2) Studies evaluated the association between sleep and MS. 3) Studies provided adjusted estimates of the relative risk (RR) (e.g., hazard ratio and odds ratio) and 95% confidence intervals (CIs). 4) Cohort studies of 1 year or longer that investigated the association between sleep and MS.

The following information was extracted from each study: 1) Name of the first author. 2) Year of publication. 3) Cohort name and country. 4) Baseline study dates. 5) Follow-up years. 6) Type of sleep disorder. 7) Sleep quality assessment tool. 8) Sleep duration. 9) Sample size. 10) Sex ratio and age. Two authors independently assessed the articles for compliance with the inclusion criteria and resolved disagreements through discussion.

### 2.3 Evaluation of Study Quality

The Newcastle Ottawa Scale (NOS) was used to assess the studies’ quality. The quality for each study was independently rated by two researchers. Due to the nature of observational studies, all the studies received 0 out of 9 points in the treatment quality. The evaluation of study quality includes eight domains: representation of the exposed cohort, selection of the non-exposed cohort, determination of exposure, absence of outcome events before the study began, whether the cohort was controlled for confounding factors, assessment of outcome events, adequacy of follow-up time, and completeness of follow-up. Each identified disagreement was resolved through team discussion and joint review.

### 2.4 Statistical Analysis

All reported RR were examined and extracted into two groups, the crude model and the most-adjusted model. The crude model is the model that does not control for covariates. The most-adjusted model is the model with the most covariates. If the study did not report the crude model, but reported the least-adjusted model and the most-adjusted model, we then pulled the least-adjusted results into the crude-model group. Hazard ratio (HR) and odds ratio (OR) were assumed to approximate the same relative risk and were collectively described as the RR in this meta-analysis. The relationship between sleep duration and MS was estimated by the combined RR and 95%CI, which were logarithmically converted. The significance of combined RR was determined by the Z-Test (P < 0.05 was considered statistically significant). The Q test and I^2^ were used to examine the heterogeneity between studies. A random-effect model (DerSimoniane-Laird method) was used to calculate pooled RR in the presence (p ≤ 0.10) or absence (p > 0.10) of heterogeneity, respectively. Subgroup analyses were conducted by the stratification of age and the components of MS. Sensitivity analyses were conducted by omitting a single study in each turn to test the robustness of our results.

Sensitivity analysis was performed after each exclusion to assess the stability of the results. Publication bias was evaluated by funnel plot, Begg’s test and Egger’s test (P < 0.05 was statistically significant). Statistical analysis was performed using Stata Version 16 (StataCorp LP, College Station, Texas, USA).

## 3 Results

### 3.1 Characteristics of the Studies

**Figure 1:**
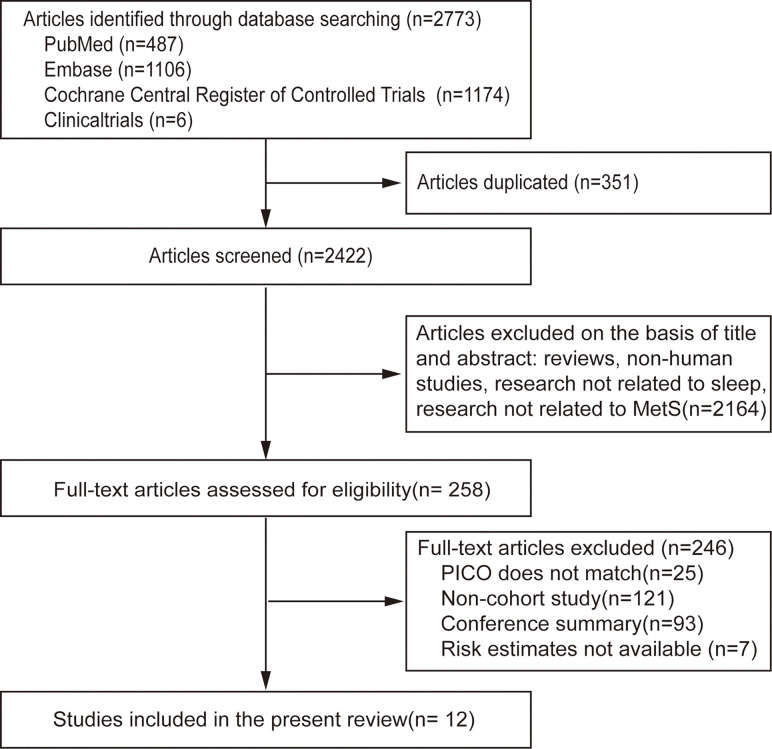
Flow chart of meta-analysis for exclusion/inclusion of individual articles.

A flow chart of meta-analysis for exclusion/inclusion of individual articles is presented in **Figure 1**. Two thousand seven hundred and seventy-three articles were identified, of which 258 (9.3%) included data for the association between sleep and MS. Finally, 12 studies (11–22) were eligible based on the inclusion and exclusion criteria. Since the data were divided by sex in one study, it was considered separate studies in the subsequent data analysis (12). Therefore, 13 studies were included in the final meta-analysis.

Of the 13 included studies, 300,202 individuals were included, with 44,211 incident MS cases were observed during follow-up, and the estimated incidence of MS was 14.7%. The duration of follow-up ranged from 1 to 18 years. The age of the study participants was between 18 to 95 years. Eleven cohorts were from Asia (92% of individuals), one from the USA (1%), and one from Europe (7%). Eight studies used American Heart Association/National Heart, Lung, and Blood Institute for Asian populations (AHA/NHLBI), one National Cholesterol Education Program (NCEP) criteria, one WHO consultation, one Japanese Guidelines, one Consensus Statement Guidelines, and one Chinese Diabetes Association criteria to define MS. Sleep duration category (hr.) and other characteristics of the included studies are presented in **Table 1**. According to the Newcastle–Ottawa scales, all of the included studies had a quality score over 9, which indicated high quality (**Table S2**).

**Table 1:** Overview of the selected studies in the meta‐analysis.

First author, year *	Cohort name, country	Base- line study dates	Follow-up (years)	Type of Sleep Disorder	Seep Quality Assessment Tool	Sleep duration	Sample size	Age (year)	Female (%)	Adjustment
Arora, 2011 (11)	TheGuangzhou BiobankCohort Study, China	2003	5	sleep duration, snoring, insomnia, daytime sleepiness.	interview	<6 6-7 Referent 7-8 8-9 ≥9	29333	50-96	64.2-78.9	age, sex, education, smoking, physical activity, insomnia, hypnotics, daytime sleepiness, mental illness, alcohol, snoring, SBP, glucose, TC, TG.
Chang, 2021 (21)	Taipei City Police, China	2013	1	sleep quality and sleep duration	PSQI	<5 5-6 6-7 Referent 7-8 ≥8	796	37.36±7.73	0	age, LDL, smoking, alcohol, exercise, snoring, shift work.
Chaput, 2013 (22)	Quebec family study, USA	1978-81	6	sleep duration	questionnaire	≤6 Referent 7-8 ≥9	193	18-65	45-65	age, sex, smoking, income, alcohol, coffee intake, caloric intake, cardiorespiratory fitness.
Deng, 2017 (13)	MJ health, China	1996	18	sleep quality and sleep duration	questionnaire	<6 Referent 6-8 >8	162161	20-80	52.6	age, sex, education, marital status, smoking, alcohol, activity, BMI, WC, SBP, glucose, TC, HDL, TG.
Itani, 2017 (14)	Medical heckup, Japan	1999	7	sleep duration	questionnaire	<5 Referent ≥5	39182	42.4±9.8	0	age, mental complaints.
Kim, 2015 (15)	KoGES-ARIRANG, South Korea.	2005-08	3	sleep duration	interview	<6 Referent 6-8 8-10 ≥10	2597	40-70	63.3-69.2	age, sex, education, smoking, alcohol, calorie intake, exercise.
Li, 2015 (16)	ChiCTR-ECH-12002938, China	2008	4.4	sleep quality and sleep duration	questionnaire	<6 6-7 Referent 7-8 8-9 ≥9	4774	30-65	36.4-59	age, sex, SBP, smoking, alcohol, activity, education, psychological pressure, bad mood, stroke, CVD, mental illness, insomnia, hypnotics, sleep quality, daytime sleep, snoring, WC, FBG, TG.
Song, 2016 (17)	The Kailuan community, China	2006-07	4	sleep quality and sleep duration	questionnaire	≤5.5 6-6.5 Referent 7 7.5-8 ≥8.5	15753	47.68± 12	15.18	age, sex, sleep duration at baseline, marital status, income, education, smoking, drinking, activity, BMI, snoring, resting heart rate, stroke, myocardial infarction, cancer.
Titova, 2018 (18)	The EpiHealth cohort study, Sweden	2011	5	sleep quality and sleep duration	questionnaire	≤6h Referent 7-8 ≥9h	19691	60.8±6.5	56.6	age, sex, education, activity, smoking, alcohol.
Yang, 2016 (19)	The Dongfeng–Tongji cohort study, China	2008	5	sleep duration	questionnaire	<6 6-7 Referent 7-8 8-9 ≥9	14399	62±7.5	48.9	age, marriage, education, smoking, drinking, activity, coronary heart disease, myocardial infarction, stroke, SBP, diabetes, BMI
Ye, 2020 (20)	REACTION, China	2012	3	sleep duration	questionnaire	<6 6-7 Referent 7-8 8-9 >9	10216	56.7± 7.7	65.3	BMI, HbA1c, SBP, TG, TC, HDL, LDL, FBG, PBG
Choi, 2011 (12) (male )	Korea	2005-06	3	sleep duration	questionnaire	<6 Referent 6-8 8-10 ≥10	386	50.63 ± 2.82	0	age, BMI, smoking, alcohol, activity.
Choi, 2011 (12) (female )	Korea	2005-06	3	sleep duration	questionnaire	<6 Referent 6-8 8-10 ≥10	721	48.49 ± 4.19	100	age, BMI, smoking, alcohol, activity, menopause.

CVD, cardiovascular disease; TC, total cholesterol; SBP, systolic blood pressure; BMI, body-mass index; WC, waist circumference;HbA1c, Glycosylated hemoglobin A1c; TG, Triglycerides; TC, Total cholesterol; HDL, High density lipoprotein; LDL, Low density lipoprotein; FBG, Fasting blood glucose; PBG, Postprandial blood glucose.

### 3.2 Meta-Analysis Results

#### 3.2.1 Sleep Duration and MS

##### 3.2.1.1 Short Sleep Duration (<6 hours) and MS in the Most‐Adjusted Model

**Figure 2:**
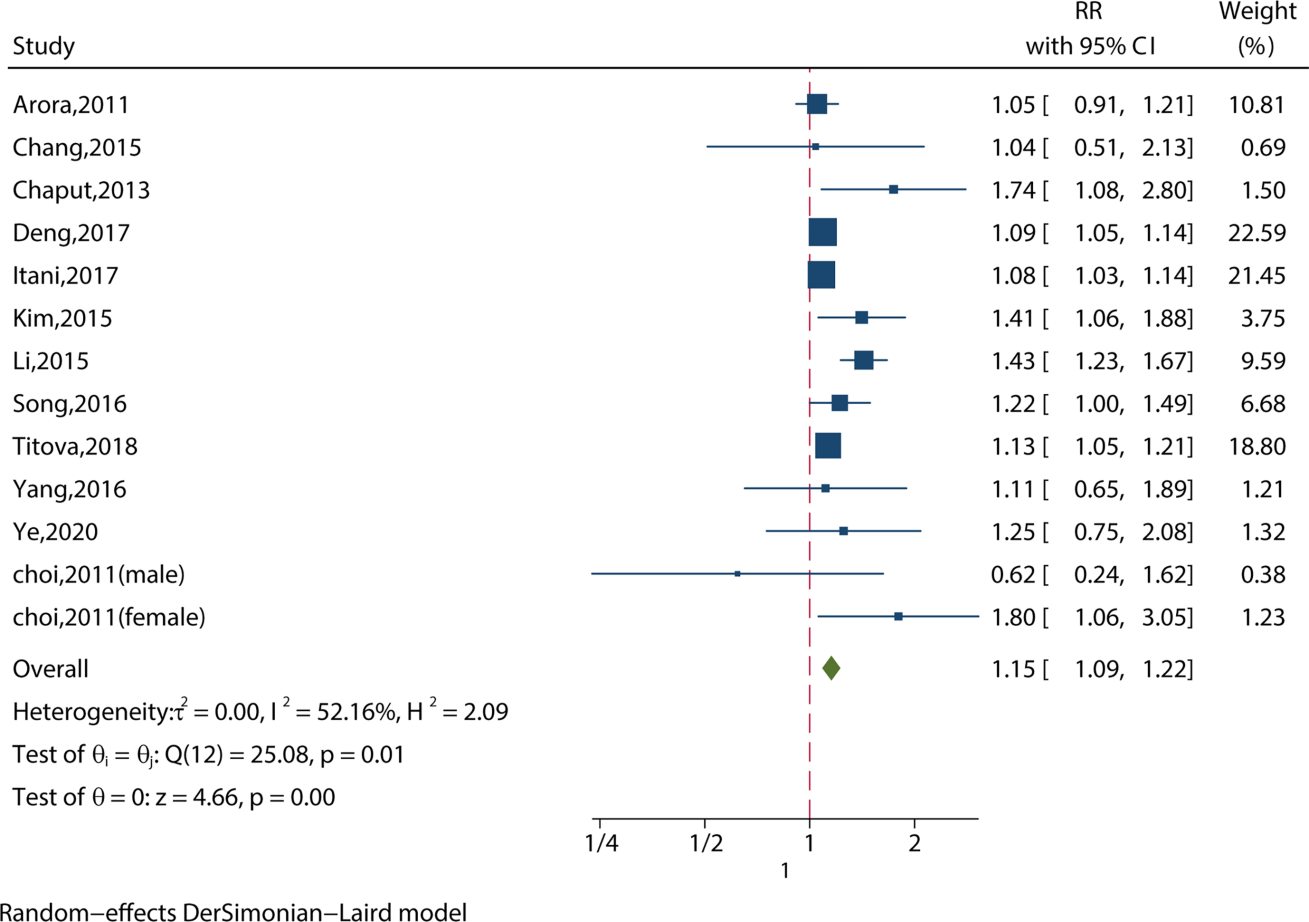
Meta-analysis of the association between short sleep and risk of metabolic syndrome in the most‐adjusted model.

We identified 13 studies reporting the RRs using the most‐adjusted model. As shown in **Figure 2**, short sleep duration increased the risk of MS significantly (RR = 1.15, 95%CI = 1.09-1.22, p < 0.001) with evidence of between-study heterogeneity (I^2^ = 52.16%, ph = 0.01). Two of the studies looked at people aged 65 or older (11, 18), and short sleep duration did not increase the risk of MS (RR = 0.98, 95%CI = 0.91-1.06, p = 0.24) without evidence of between-study heterogeneity (I^2^ = 26.22%, ph = 0.64).

##### 3.2.1.2 Long Sleep Duration (>8 hours) and MS in the Most‐Adjusted Model

**Figure 3:**
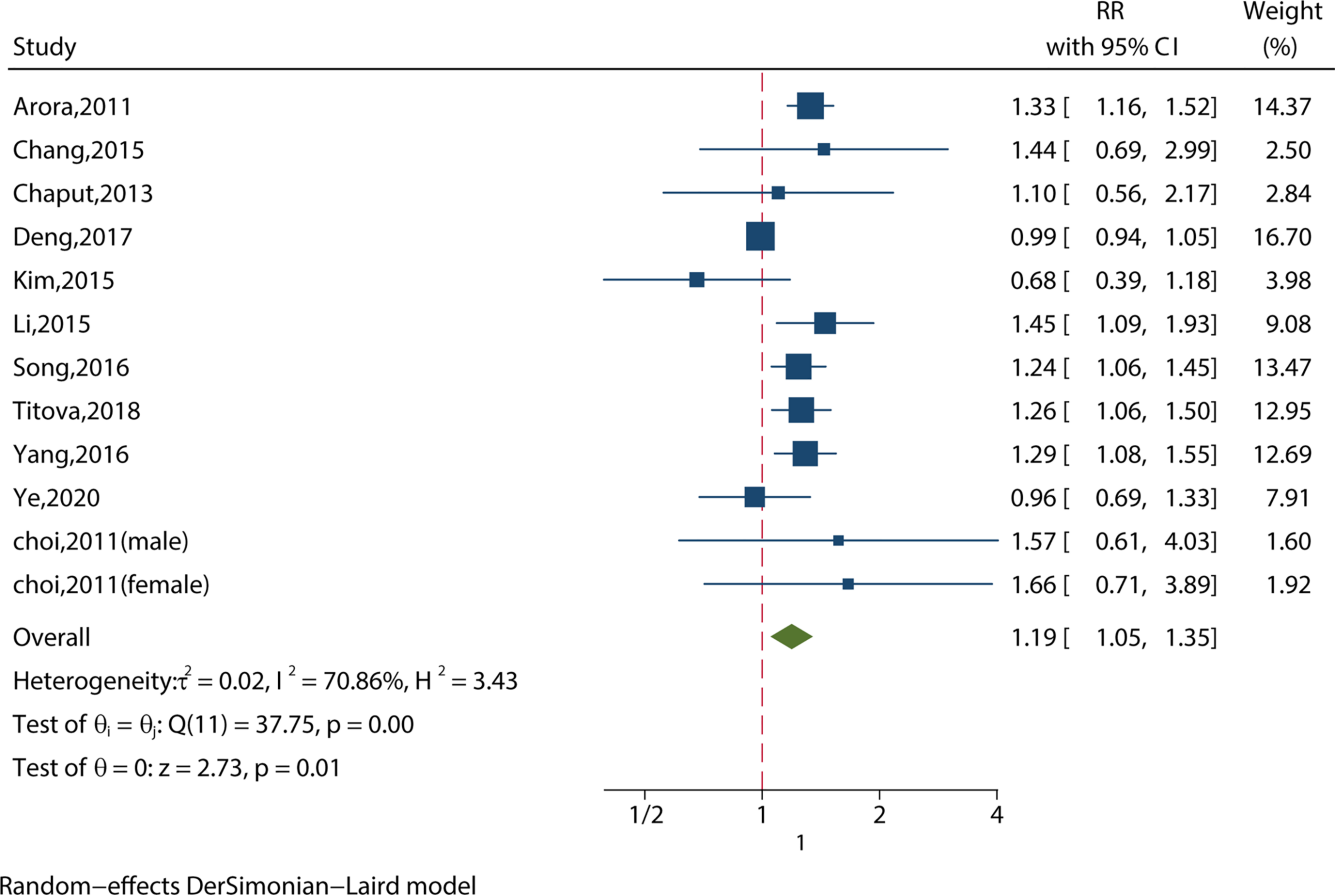
Meta-analysis of the association between long sleep and risk of metabolic syndrome in the most‐adjusted model.

We identified 12 studies reporting the RRs using the most‐adjusted model. As shown in **Figure 3**, long sleep duration increased the risk of MS significantly (RR = 1.19, 95%CI = 1.05-1.35, p < 0.001) with evidence of between-study heterogeneity (I^2^ = 70.86%, ph = 0.01). Two of the studies investigated people aged 65 or older (11, 18), and found that long sleep duration increased the risk of MS (RR = 1.19, 95%CI = 1.04-1.35, p = 0.01) without evidence of between-study heterogeneity (I^2^ = 50.38%, ph = 0.16).

##### 3.2.1.3 Short Sleep Duration (<6 hours) and MS from Crude Models (or Least‐Adjusted Models)

**Figure 4:**
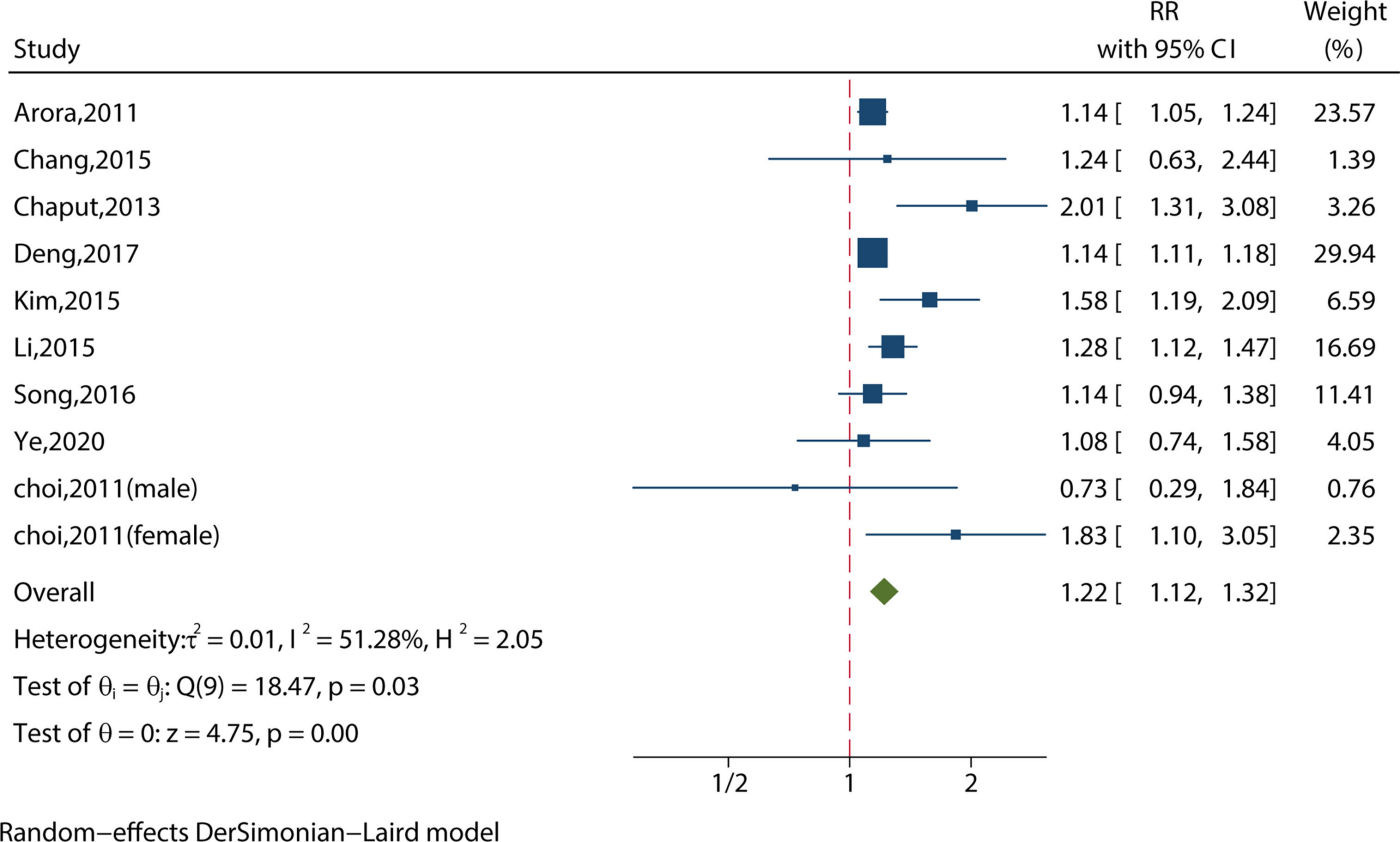
Meta-analysis of the association between short sleep and risk of metabolic syndrome from crude models or least-adjusted results.

We identified 10 studies reporting the RRs using a crude model or least-adjusted model. As shown in **Figure 4**, short sleep duration increased the risk of MS significantly (RR = 1.22, 95%CI = 1.12-1.32, p < 0.001) with evidence of between-study heterogeneity (I^2^ = 51.28%, ph = 0.03).

##### 3.2.1.4 Long Sleep Duration (>8 hours) and MS from Crude Models (or Least‐Adjusted Models)

**Figure 5:**
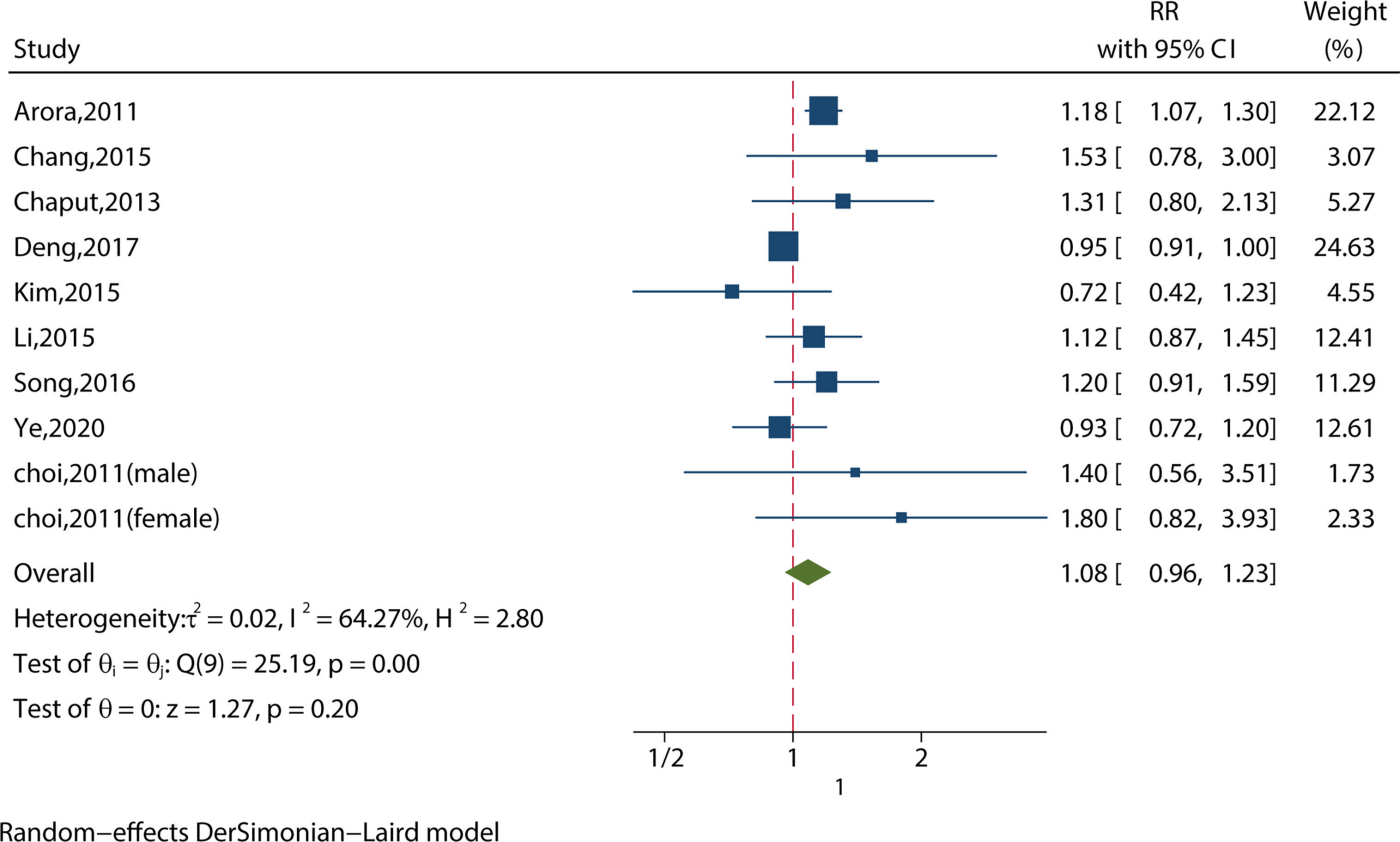
Meta-analysis of the association between long sleep and risk of metabolic syndrome from crude models or least-adjusted results.

We identified 10 studies reporting the RRs using a crude model or least-adjusted model. As shown in **Figure 5**, long sleep duration did not increase the risk of metabolic syndrome (RR = 1.08, 95%CI = 0.96-1.23, p = 0.20) with evidence of between-study heterogeneity (I^2^ = 64.27%, ph = 0.001).

##### 3.2.1.5 A U-shaped Relationship Between Sleep Duration and Metabolic Syndrome.

Sleep duration (6-7 hours) increased the risk of MS (RR = 1.08, 95%CI = 1.01-1.16, p = 0.03; I^2^ = 15.03%, p = 0.32) (**Figure S1**). Sleep duration between 8 and 9 hours increased the risk of MS (RR = 1.12, 95%CI = 1.01-1.24, p = 0.04; I^2^ = 52.17%, p = 0.10; **Figure S2**). Sleep duration with more than 9 hours) increased the risk of MS (RR = 1.25, 95%CI = 1.13-1.39, p < 0.001; I^2^ = 19.27%, p = 0.27) (**Figure S3**).

**Figure 6:**
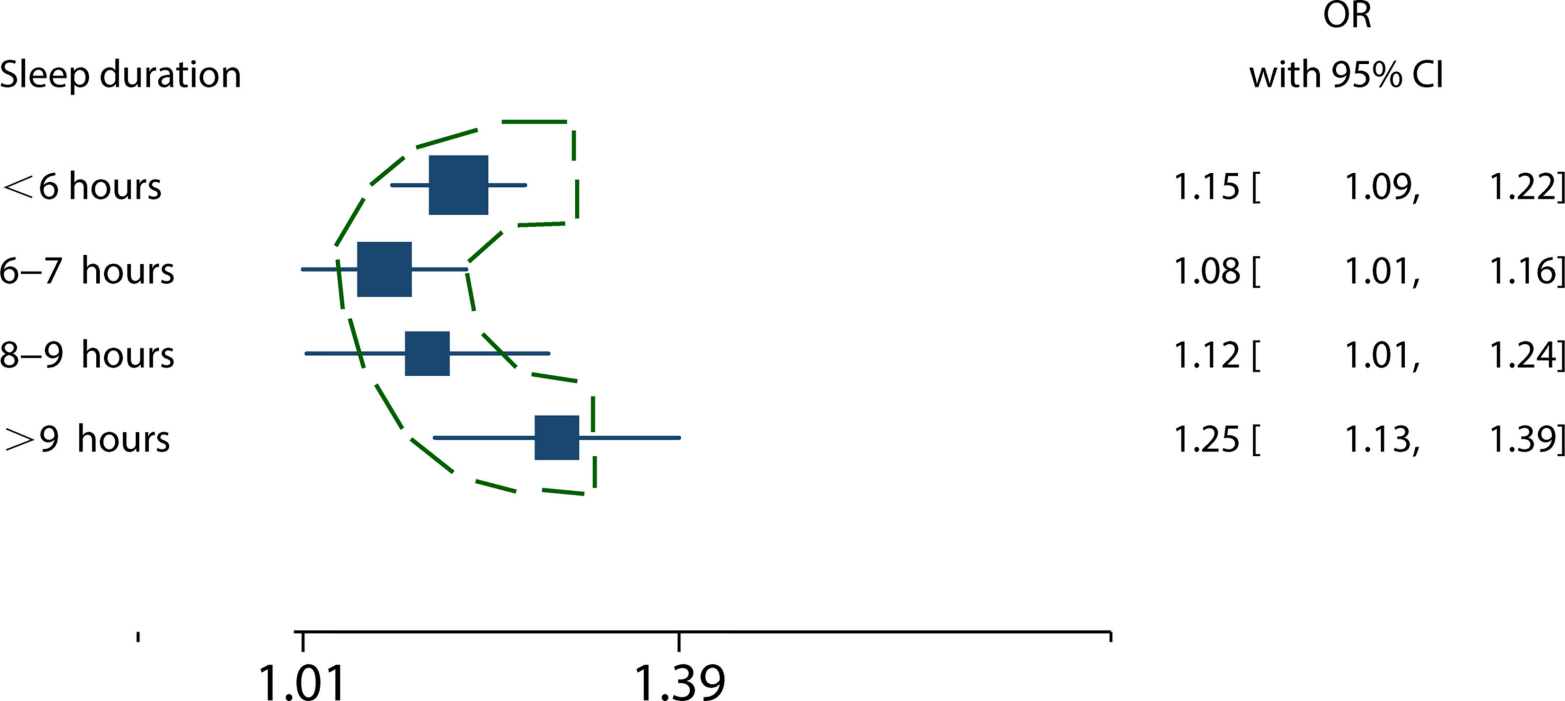
The relationship between sleep duration and MS risk presented a U-shaped curve. The squares represent the mixed RRs of different sleep duration in patients with MS. The broken outline in green illustrates the nonlinear trend, indicating the presence a U-shaped relationship.

Summary forest plot with a comparison of pooled RRs for different durations (*<6 hours*, 6-7 hours, 8-9 hours, and >9 hours) of sleep with MS risk presented a U-shaped curve (**Figure 6**).

#### 3.2.2 Sleep Duration With Components of MS

##### 3.2.2.1 Short Sleep Duration With Components of MS

The relationship between short sleep duration and components of the metabolic syndrome are presented in **Table 2**. Short sleep duration increased the risk of obesity (14% (95%CI=7-22%, p<0.001; I^2^ = 48.91%, ph = 0.07), hyperglycemia (RR = 1.12, 95%CI = 1.00-1.15, p = 0.05; I^2^ = 78.32%, p = 0.001) and hypertension (16% (95%CI=2-31%, p = 0.03; I^2^ = 90.56%, ph<0.001). However, short sleep duration does not increased the risk of hypertriglyceridemia (RR = 1.04, 95%CI = 0.96-1.12, p = 0.37; I^2^ = 65.06%, p = 0.01) and low HDL (RR = 0.98, 95%CI = 0.88-1.23, p = 0.11; I^2^ = 52.78%, p = 0.06).

**Table 2:** Meta-analysis of the associations between sleep duration and the components of MS.

	Study number	RR	95%CI	P _Z_	Statistical model	I^2^ (%)	P _H_
Short sleep							
Obesity	7	1.14	1.07-1.22	<0.001	Random	48.91	0.07
Hyperglycemia	7	1.12	1.00-1.25	0.05	Random	78.32	<0.001
Hypertension	7	1.16	1.02-1.31	0.03	Random	90.56	<0.001
Hypertriglyceridemia	7	1.04	0.96-1.12	0.37	Random	65.06	0.01
Low HDL	6	0.98	0.88-1.09	0.73	Random	46.39	0.10
Long sleep							
Obesity	6	1.15	1.01-1.30	0.04	Random	57.7	0.04
Hyperglycemia	6	1.08	0.88-1.31	0.47	Random	81.32	<0.001
Hypertension	6	1.13	1.04-1.24	0.01	Random	52.36	0.06
Hypertriglyceridemia	6	1.22	0.98-1.52	0.07	Random	83.85	<0.001
Low HDL	6	1.10	0.98-1.23	0.11	Random	52.78	0.06

RR, relative ratio; CI, confidence interval.

P_Z_, P value for Z test.

P_H_, P value based on Q test for between-study heterogeneity.

##### 3.2.2.2 Long Sleep Duration With Components of MS

The relationship between long sleep duration and components of the metabolic syndrome are presented in **Table 2**. Long sleep duration increased the risk of obesity (RR = 1.15, 95%CI = 1.01-1.30, p = 0.04; I^2^ = 57.70%, p = 0.04), hyperglycemia (RR = 1.08, 95%CI = 0.88-1.31, p = 0.47; I^2^ = 81.32%, p< 0.001)and hypertension (RR = 1.13, 95%CI = 1.04-1.24, p = 0.01; I^2^ = 52.36%, p = 0.06). However, long sleep duration did not increase the risk of hypertriglyceridemia (RR = 1.22, 95% CI = 0.98-1.52, p = 0.07; I^2^ = 83.85%, p< 0.001) and low HDL (RR = 1.10, 95%CI = 0.98-1.23, p = 0.11; I^2^ = 52.78%, p = 0.06).

### 3.3 Sensitivity Analysis and Publication Bias

After excluding one study at a time, the sensitivity analysis confirmed the increased risk of MS for short sleep duration [RR with 95%CI ranging from 1.16 (1.09-1.23) to 1.18 (1.10-1.26)]. Similarly, the significant statistical significance was confirmed by the sensitivity analysis for long sleep duration (RR with 95%CI ranging from 1.18 (1.04-1.35) to 1.22 (1.07-1.39)). No publication bias was detected for short sleep duration (Begg’s test: p = 0.591 and Egger’s test: p = 0.102) and long sleep duration (Begg’s test: p = 1.369 and Egger’s test: p = 0.785). A visual inspection of the funnel plots also did not reveal apparent publication bias (**Figure S4**).

## 4 Discussion

In this study, we examined the causal relationship between sleep duration and MS by enrolling cohort studies and using meta-analysis techniques. Our findings suggest that both short and long sleep periods result in an increased risk of MS, and the risk of longer sleep duration is higher than that of short sleep duration. Moreover, the duration of sleep and the risk of MS presented a U-shaped curve. Both short and long sleep periods can increase the risk of MS components, such as obesity and high blood pressure. Short periods of sleep may increase the risk of hyperglycemia, another component of MS. A large sample size of 300,202 participants and the absence of publication bias make our study results more robust. Sensitivity analysis further confirmed the robustness of our conclusion.

Cohort studies move towards results and the temporal sequence between causes and effects is usually clear, whereas cross-sectional studies are difficult to make causal inferences or interpret established associations (23). Long sleep duration has not been found to be associated with an increased risk of MS in previous research (7, 24), which was mostly based on cross-sectional studies (25). Our findings show that both long and short sleep duration are associated with an increased risk of MS. The simplest model and the one with the most adjustments were derived from our meta-analysis. Surprisingly, long sleep had the opposite effect on MS, with the estimated effect in the crude model being non-statistically significant but evident in the adjusted model. This may be the reason why previous systematic evaluations could not yield positive results.

Interestingly, we found that short sleep did not increase the risk of MS in persons older than 65 years. It appears that the amount of sleep required by the elderly is less than that required by the young (26). First, older people slept significantly less than younger people when extremely long mandatory sleep periods were provided (27). This may be due to the fact that older people have lower ad lib sleep needs than younger people. Another interesting finding was that in healthy older adults, after sleep deprivation or experimental slow-wave sleep suppression, slow-wave sleep duration and slow-wave activity (SWA) rebounded more slowly than in younger adults (28, 29). Older people have lower homeostatic sleep buildup than younger people, based on this finding. Another finding is that older people have less subjective and objective sleepiness after selective slow-wave sleep deprivation, even when they’re resting (28). In addition, under sleep deprivation, the elderly showed less impairment in the sleep sensitive alert task than young people (30). There are numerous phenotypic signs of reduced homeostatic sleep drive in older people, which suggests that they require less sleep (27, 28). In other words, as we get older, our sleep requirements become less.

One literature only showed a dose-response relationship between short sleep duration and MS (8). Our meta-analysis collected evidence for further analysis of different sleep duration categories (<6 hours, 6-7 hours, 8-9 hours, and >9 hours). Our meta-regression results showed a U-shaped prediction of dose-dependent responses, which had not been found in previous meta-analyses. In other words, compared to seven to eight hours of sleep, every one-hour decrease in sleep duration corresponds to an 8% increase of the MS risk. Moreover, the risk of MS rises by 12% for each additional one hour of sleep.

Besides, we investigated the relationship between sleep duration and components of MS. Short sleep duration and long sleep duration increased the risk of obesity by 14% and 15%, respectively; the risk of hypertension was increased by 16% and 13%, respectively. Short sleep duration potentially increased the risk of hyperglycemia by 12%, while long sleep duration did not increase the risk of hyperglycemia. The possible reason is that the impaired blood glucose in MS mainly refers to elevated fasting blood glucose. However, many patients with impaired blood glucose present with normal fasting blood glucose and elevated postprandial blood glucose (31). For low-HDL, another component of MS, there was no statistically significant difference between short and long sleep duration.

The underlying mechanisms of the relationship between sleep and MS are not fully understood. The underlying mechanisms linking sleep to MS may differ between short and long term sleep. Several potential pathophysiology mechanisms may contribute to the relationship between short sleep duration and MS. Hormonal changes may be part of the explanation for MS caused by a short sleep. The low level of promoting anorexia hormone leptin and higher hunger hormone ghrelin levels has been found during short sleep in some experimental research (32, 33). Other hormone changes caused by the short sleep periods include an increase in cortisol production at night (34–36), a kind of hormone that can cause insulin resistance and promote weight gain, hyperglycemia and hypertension. Elevated catecholamines lead to increased sympathetic nerve activity, endothelial cell dysfunction, and impaired vasodilation. These changes also contribute to increased blood pressure (37–41). Some short sleep was found to reduce insulin sensitivity in adipocytes and affect the phosphorylation of the serine/threonine kinase Akt in the insulin signaling pathway (42).

The underlying mechanism between longer sleep and an increased risk of MS is currently thought to be speculative. Obstructive sleep apnea (OSA) may be a cause. Risk factors for OSA include snoring, increased body mass index (BMI), and aging. Snoring patients begin sleep with reduced pharyngeal dilator activity, leading to apnea and hypoventilation, and end with a slight awakening that restores muscle activity and reopens the upper airway. Repeated episodes of this can cause sleep fragmentation, which is associated with long periods of sleep. Sleep fragmentation and intermittent hypoxia can increase the excitability of the sympathetic nervous system, resulting in metabolic disorders (43). Moreover, About 70% of OSA patients are obese, which is a component of metabolic syndrome and is associated with insulin resistance. One study showed that weight loss in moderate to severe OSA patients reduced upper respiratory tract collapse and daytime sleepiness (44). Older adults have ventilatory control systems that are out of control. At the same time, older people have more airway collapse and lower ventilation capacity than younger people, and longer sleep associated with OSA makes them more vulnerable to metabolic disorders due to hypoxia (45). Finally, long sleep duration has been associated with several risk factors for MS morbidity, such as depression and low physical activity (46).

In addition, sleep appears to promote inflammatory homeostasis by affecting a variety of inflammatory mediators, such as cytokines. Prolonged sleep disturbances can lead to chronic, systemic, low-grade inflammation and are associated with a variety of diseases with inflammatory components, such as MS. This view is supported by Besedovsky (47). A consequence of MS as a chronic, low-grade inflammation is insulin resistance (48, 49). Various measures associated with the innate immune system have been used to assess low-grade inflammation, including C-reactive protein (CRP), IL-6, WBC count, neutrophil count, and platelet count (50). Studies have shown that short sleep duration is combined with increased inflammatory load (i.e., CRP, IL-6, TNF, and IFN-γ levels) (51). However, many studies have also reported the association between elevated inflammatory markers and long sleep duration (52–54). Although statistical adjustments have been made for various factors, including demographic variables, health behaviors, medication, and comorbidities, the association between long-term sleep duration and elevated inflammatory status is thought to be mediated by uncontrolled disease processes.

It should be noted that the results of this meta-analysis are based on self-reported sleep duration. Some researchers believe there is a common “sleep state misperception “ among people, which means they underestimate how long they sleep. Fernandez-Mendoza found sleep state misperception are present in people with normal sleep duration, but not in people who sleep less. In addition, sleep state misperception was associated with depression, anxiety-reflective personality traits and poor coping resources, but not with other factors, such as gender, age, race and education (55). In addition, different measurement tools of sleep duration may affect the interpretation of the study results and subsequent clinical application (56). Polysomnography (PSG) is the gold standard for measuring sleep, but its use is limited by cumbersome procedures. Matthews (57) proved that self-reported sleep duration was about 20-30 minutes longer than PSG. The sleep estimates assessed by PSG were 7 to 20 minutes longer than sleep duration assessed by activity-recording (57). Self-reported sleep duration is more widely used because it usually consists of a simple retrospective questionnaire given. Notably, self-reported habitual sleep duration was moderately associated not only with PSG sleep duration, but also with activity recording. The overlap variances are 16% and 25-29%, respectively (57, 58). Additionally, there was some heterogeneity in the results. Definitions of MS definitions are inconsistent, although similar in most respects, which may cause some heterogeneity in our results. Finally, the included studies were mainly conducted in Asia, the United States, and Sweden. These findings may not generalize to other ethnic groups.

## 5 Conclusion

After considering the typical risk factors for MS, both short and long sleep periods can increase the risk of MS. Furthermore, the relationship between the dose of sleep duration and MS risk presented a U-shaped curve. For the components of MS, both short and long sleep increased the risk of obesity and high blood pressure. Short sleep can potentially increase the risk of high blood sugar. However, the exact mechanism that causes this difference is not clear. Clinically doctors and health professionals should be encouraged to increase their efforts to promote healthy sleep for all people. Current trends in sleep disorders indicate the critical importance of integrating healthy sleep into MS control policies. Longer-term randomized controlled trials are needed to establish causality and to elucidate the underlying mechanisms.

## Data Availability Statement

The original contributions presented in the study are included in the article/**Supplementary Material**. Further inquiries can be directed to the corresponding authors.

## Author Contributions

Conception and design: ZW, XL, and TC. Data collection and interpretation: TC, CY, and DT. Data analyses: XZ. Manuscript draft and critical review: all authors. Final approval of the study content and manuscript and accountability for data integrity: all authors.

## Funding

This work was supported by the National Natural Science Foundation of China (No. 81671835 and No. 32171339), the Key Projects of Tianjin Natural Science Foundation (No. 19JCZDJC36900), and the Tianjin Commission of Science and Technology (No. RC20165 and No. ZC20143).

## Conflict of Interest

The authors declare that the research was conducted in the absence of any commercial or financial relationships that could be construed as a potential conflict of interest.

## Publisher’s Note

All claims expressed in this article are solely those of the authors and do not necessarily represent those of their affiliated organizations, or those of the publisher, the editors and the reviewers. Any product that may be evaluated in this article, or claim that may be made by its manufacturer, is not guaranteed or endorsed by the publisher.
