# Supplementary material for: The Association Between Sleep and Metabolic Syndrome: A Systematic Review and Meta-Analysis
Source: Front Endocrinol (Lausanne). 2021 Nov 19;12:773646. doi: 10.3389/fendo.2021.773646 (PMC8640251; doi:10.3389/fendo.2021.773646)

Supplementary materials: Table 1s: search strategy
PubMed
#1 Sleep [Mesh]
#2 Sleep disorders [Mesh]
#3 sleep*[Title/Abstract]
#4 insomnia*[Title/Abstract]
#5 wakeful*[Title/Abstract]
#6 dyssomn*[Title/Abstract]
#7 "Sleep Initiation and Maintenance Disorders" [Mesh]
#8 "disorders of excessive somnolence"[Mesh]
#9 hypersomnia[Title/Abstract]
#10 parasomnia[Title/Abstract]
#11 circadian*[Title/Abstract]
#12 "rest activity" [Title/Abstract]
#13 somnolence[Title/Abstract]
#14 sundowning[Title/Abstract]
#15 "Chronobiology Disorders"[Mesh]
#16 "Circadian Rhythm"[Mesh]
#17 vigilance[Title/Abstract]
#18 alertness[Title/Abstract]
#19 drowsiness[Title/Abstract]
#20 hypersomnolence*[Title/Abstract]
#21 eveningness[Title/Abstract]
#22 morningness[Title/Abstract]
#23 arousal[Title/Abstract]
#24 "Psychomotor Performance"[Mesh]
#25 fatigue[Title/Abstract]
#26 "neurocognitive performance"[Title/Abstract]
#27 "concentration difficulties"[Title/Abstract]
#28 OR/ #1-#27
#29 Metabolic syndrome[MeSH]
#30 Metabolic syndrome[Title/Abstract]
#31 syndrome x[Title/Abstract]
#32 metS[Title/Abstract]
#33 insulin resistance syndrome[Title/Abstract]
#34 "Metabolic X Syndrome"[Title/Abstract]
#35 "Metabolic Cardiovascular Syndrome"[Title/Abstract]
#36 cardio-metabolic syndrome[Title/Abstract]
#37 OR/ #29-#36
#38 #28 AND #37

Table 2s. Newcastle-Ottawa Quality Assessment Scale of the cohort studies.

Study (First author/year)	**Selection**				Comparability	**Outcome**			Total scores
	Representative-ness of the exposed cohort	Selection of the non-exposed cohort	Ascertainment of exposure	Demonstration that outcome of interest was not present at start of study		Assessment of outcome	Was follow-up long enough for outcomes to occur	Adequacy of follow up of cohorts	
Arora,2011	★	★	★	★	★★	★	★	★	9
Chang,2015	★	★	★	★	★★	★	★	★	9
Chaput,2013	★	★	★	★	★★	★	★	★	9
Deng,2017	★	★	★	★	★★	★	★	★	9
Itani,2017	★	★	★	★	★★	★	★	★	9
Kim,2015	★	★	★	★	★★	★	★	★	9
Li,2015	★	★	★	★	★★	★	★	★	9
Song,2016	★	★	★	★	★★	★	★	★	9
Titova,2018	★	★	★	★	★★	★	★	★	9
Yang,2016	★	★	★	★	★★	★	★	★	9
Ye,2020	★	★	★	★	★★	★	★	★	9
choi,2011	★	★	★	★	★★	★	★	★	9

Fig. 1s. Meta-analysis of the association between sleep duration (6-7 hours) and risk of metabolic syndrome.

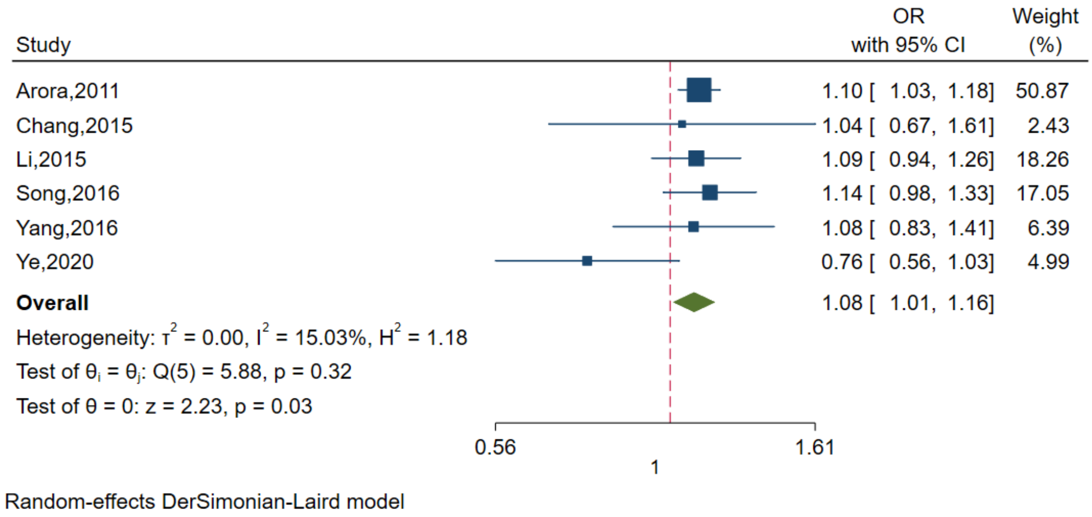

Fig. 2s. Meta-analysis of the association between sleep duration (8-9 hours) and risk of metabolic syndrome.

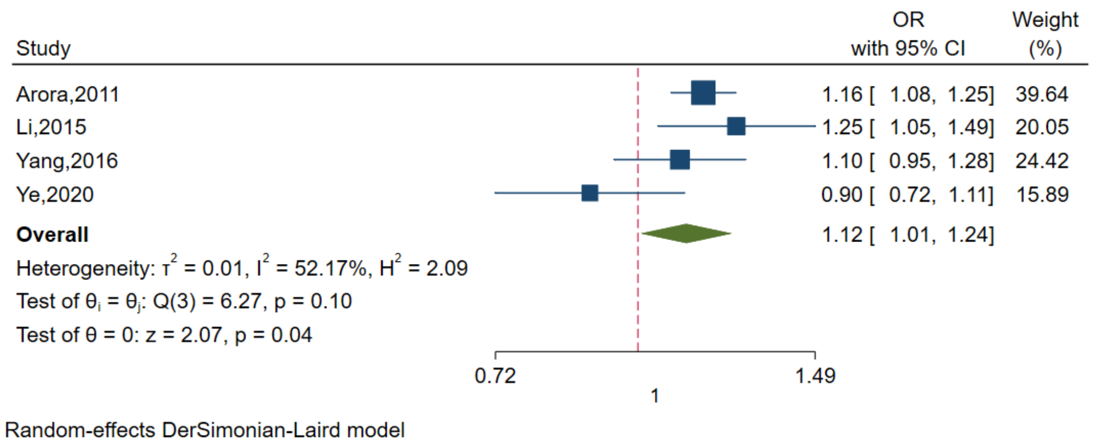

Fig. 3s. Meta-analysis of the association between sleep duration (＞9 hours) and risk of metabolic syndrome.

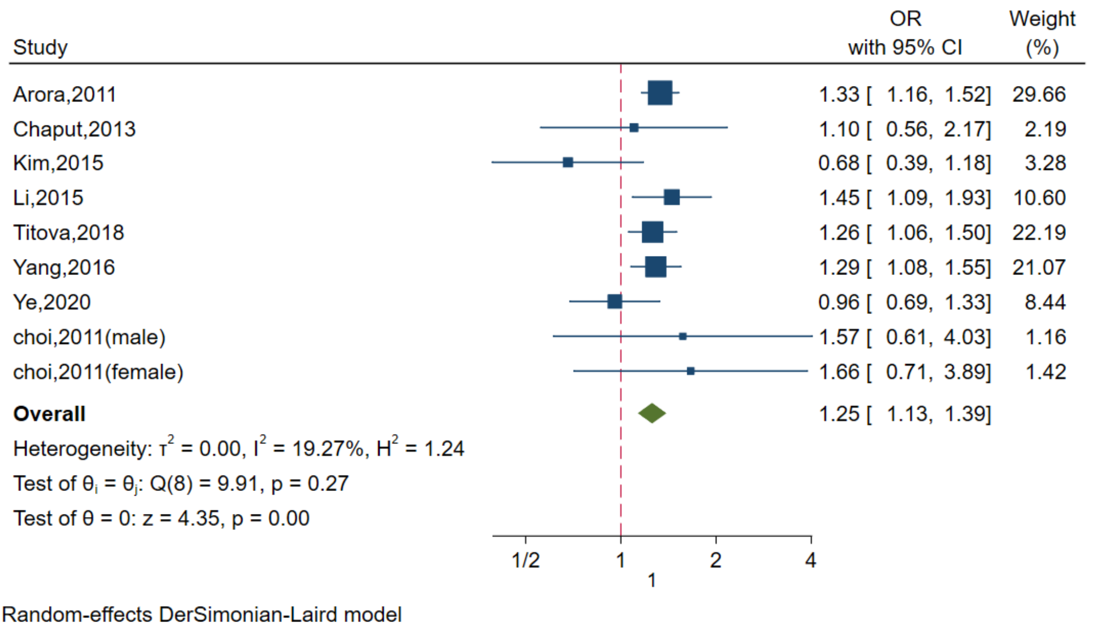

Fig. 4s. Funnel plot

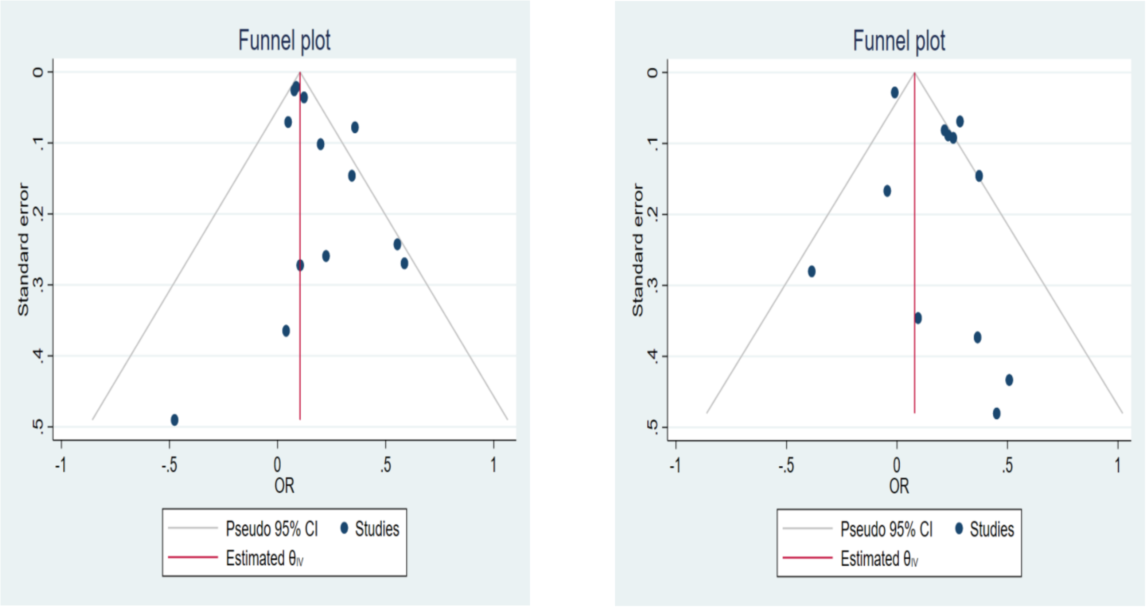

Supplement: Supplementary file 1 [file DataSheet_1.pdf]
